# Factors associated with adherence to iron folate supplementation among pregnant women in West Dembia district, northwest Ethiopia: a cross sectional study

**DOI:** 10.1186/s13104-019-4045-2

**Published:** 2019-01-06

**Authors:** Tsegaye Molla, Tadesse Guadu, Esmael Ali Muhammad, Melkamu Tamir Hunegnaw

**Affiliations:** 1 Amhara Region Central Gondar Health Department Northwest Ethiopia, University of Gondar, P.o.box.196, Gondar, Ethiopia; 2 Department of Environmental and Occupational Health, College of Medicine and Health Sciences, University of Gondar, P.o.box.196, Gondar, Ethiopia; 3 Department of Human Nutrition, Institute of Public Health, College of Medicine and Health Sciences, University of Gondar, P.o.box.196, Gondar, Ethiopia

**Keywords:** Adherence, Iron folate supplementation, Pregnant women, Ethiopia

## Abstract

**Objective:**

In Ethiopia, iron folate tablets are prescribed for all pregnant mothers during their antenatal visits and given for free; however, only limited data are available on their adherence. Therefore, the aim of this study was to assess adherence to iron folate supplementation and its associated factors among pregnant women in West Dembia district, northwest Ethiopia. An institution based cross-sectional study was conducted on 348 pregnant women that had at least one antenatal care visit. Bivariate and multivariate logistic regressions were employed to identify the predictors at p-value < 0.2 and 0.05 respectively.

**Results:**

Adherence to iron folate supplementation in this study was 52.9% [95% CI (47.7, 58.0%)]. Women who had good knowledge about anemia (AOR: 2.63, 95% CI 1.51, 4.59), knowledge about iron folate supplementation (AOR: 2.82, 95% CI 1.52–5.23), four and more ANC visits (AOR: 6.97, 95% CI 3.25, 14.96), and anemia history during current pregnancy (AOR: 13.87, 95% CI 3.75, 51.35) were significantly associated with adherence to iron folate supplementation. Therefore, preventing prenatal anemia, improving knowledge of women about anaemia and iron folate supplementation, and increasing ANC services are essential to increase adherence.

## Introduction

Globally, 41.8% of pregnant women are anemic, half of the burden being due to iron deficiency [1]. Iron deficiency anemia aggravates maternal blood loss and infections at childbirth; and it is also associated with increased prenatal mortality and morbidity contributing to low birth weight, lowered resistance to infections, poor cognitive development, reduced work capacity, and a significant impact on economic growth and development [2, 3].

Iron deficiency anemia, which occurs due to an increased requirement of iron folate, is common during pregnancy [4]. Supplementation has been a major strategy in low and middle-income countries to reduce iron deficiency anemia during pregnancy. The recommended daily dose is 30–60 mg of elemental iron and 400 μg of folic acid [5]. A study suggested that iron folate supplementation would increase the mean hemoglobin concentration of pregnant women by 10.2 g/l by eliminating about 50% of anemia [6].

Even though, pregnant women in Ethiopia take a daily oral iron folate supplements as part of the ANC, the prevalence of anemia increased from 22% in 2011 to 29% in 2016 [7]. WHO and the Ethiopian National Nutrition Program recommend iron folate tablets for all pregnant women, but the magnitude of adherence is still within the range of 20.4% [8] to 70% [9], which is quite low.

The variation and magnitude of adherence is influenced by multiple factors, like socio-demographic, ANC visits, nutrition counseling, and knowledge about anemia as well as iron folate supplementation [10–12]. Improving adherence is therefore essential for eliminating anaemia and to make iron supplementation programs successful. Therefore, this study was done to determine adherence and associated factors relating to iron folate supplementation among pregnant women in Dembia district northwest, Ethiopia.

## Main text

### Methods and materials

#### Study setting

A facility based cross-sectional study was conducted to determine the adherence and associated factors of iron folate supplementation among pregnant women in West Dembia district, northwest Ethiopia, 2018. The district is located in Central Gondar Zone, Amhara Region about 245 km from Bahir Dar. The total population of the district is 131, 412 of which 23.58% are in the reproductive age group. Nearly, 4428 women are expected to get pregnant within a year. The district has five public health centers which give comprehensive ANC and maternal health care services.

#### Sample size and sampling method

The sample size was calculated by using the single population proportion formula. The magnitude of adherence to iron folate supplementation was 28.9% [13], with a precision of 5% and 95% CI. The sample size 316, became 348 after adding, a 10% non response rate.

All health centers in the district were included in the study, and the number of participants per health center was allocated based on the average number of pregnant women who visit each health center per month. Previous ANC follow ups per month for Abawram, Abrjha, Chuhait, Gorgora, Sankisa health centers were 161, 128, 266, 26, and 185, respectively. Based on this data and the sample size of the study, the participants of each health center were selected using a systematic random sampling method with intervals of two.

#### Data collection and analysis

The data collection instruments were close-ended questions, and questionnaire contiens included socio-demographic, obstetric history, knowledge about anemia, and iron folate supplementation related variables. The pregnant women who took iron folate tablets at least 4 days in recent weeks were considered to have adhered to the supplementation [14].

The questionnaire was pretested on 34 pregnant women of similar population in East Dembia district, Koladiba health center. Data were collected and supervised by five diploma and two BSc nurses degree graduate nurses, respectively. Both data collectors and supervisors were trained on how to collect the data and how to use the data collection instruments. The supervisors checked the completeness of the data every day.

The collected data were entered, coded and cleaned, using Epi INFO version 7.0, and data management and analyses were performed using SPSS version 20.0 software. Socio-demographic, obstetric, and medical history of pregnant women were presented in texts and tables. Bivariate analysis was done and variables with less than 0.2 p-values were included in the multiple logistic regression analysis. In the multivariate analysis, predictors with p < 0.05 were considered statistically significant.

### Results

#### Socio-demographic characteristics of the study subjects

A total of 348 pregnant women that had at least one antenatal care visit were interviewed with a response rate of 100%. The mean age of the participants was 26.86 years (± 5.78 standard deviations) with a minimum and maximum of 18 and 46 years respectively. Two hundred seventy-one (77.9%) were rural dwellers. Most of the participants, 339 (97.4%), were Orthodox Christians and 334 (96%) were married. Regarding educational status, 193 (55.5%) were unable to read and write. One hundred thirty-six (39.1%) and 134 (38.5%) were farmers and housewives, respectively (Table 1).

**Table 1:** Socio- demographic characteristics of pregnant women in West Dembia northwest Ethiopia 2018 (N = 348)

Characteristics	Category	Frequency	Percentage
Age in years	15–24	117	33.6
	25–34	189	54.3
	≥ 34	42	12.1
Religion	Orthodox	339	97.4
	Muslim	9	2.6
Residence	Rural	271	77.9
	Urban	77	22.1
Educational status	Unable to read and write	193	55.5
	Able to read and write	69	19.8
	Elementary completed	34	9.8
	Secondary completed	27	7.8
	College and above	25	7.2
Marital status	Married	334	96.0
	Single	14	4.0
Monthly income in Ethiopian Birr	< 1000	31	89.0
	1000–3000	258	74.1
	> 3000	59	17.0
Current occupation	Farmer	136	39.1
	Housewife	134	38.5
	Daily laborer	10	2.9
	Merchant	32	9.2
	Government employee	33	9.5
	Privet employee	3	0.9

#### Adherence to iron folate supplementation

In this study, adherence to iron folate supplementation among pregnant women was 52.9% with 95% CI (47.7, 58.0).

#### Health and health service related characteristics

Most of the participants, 262 (75.3%), were multi gravid, and 62 (17.8%) pregnant women had four and above ANC visits during pregnancy. Only 10 (2.9%) of the participants had hypertension in the current pregnancy, 21 (6%) infected by malaria, and 2 (0.6%) had other complications. About 57.5% and 68.7% of the participants had good knowledge about anemia and good iron folate supplementation, respectively. The main reasons for non-adherence to iron folate supplementation were forgetfulness (41%), fear of side effect (40%), and fear of increased baby size (29%) (Table 2).

**Table 2:** Obstetrics and medical history of pregnant women in West Dembia northwest Ethiopia 2018 (N = 348)

Characteristics	Category	Frequency	Percentage
Gravidity	Prim gravid	262	75.3
	Multi gravid	86	24.6
Number of ANC visit	≤ 2	163	46.8
	3	123	35.3
	≥ 4	62	17.8
History of hypertension during current pregnancy	Yes	10	2.9
	No	338	97.1
History of anaemia during current pregnancy	Yes	31	8.9
	No	317	91.1
History of malaria attack during current pregnancy	Yes	21	6.0
	No	327	94.0
Knowledge about anemia	Poor knowledge	148	42.5
	Good knowledge	200	57.5
Knowledge about iron folate supplementation	Poor knowledge	109	31.3
	Good knowledge	239	68.7
Reasons for adherence (multiple response possible)	Advice of health worker	147	90
	Tablets would increase their blood	39	24
	Fear of illness	28	17
	Family support	24	15
	Free in charge	19	12
	Reminding technique	10	6
	Direct consumer advertising	7	4

#### Factors associated with adherence to iron folate supplementation

Binary logistic regression was carried out to determine factors associated with adherence to iron folate supplementation. In the multivariate logistic regression analysis, ANC visits, anemia before this pregnancy, prenatal knowledge about anemia, and iron folate supplementation were found to be statistically significantly associated with adherence to iron folate supplementation for pregnant women at a 95% CI and a p-value of 0.05.

According to the binary logistic regression model, the odds of adherence to iron folate supplementation 2.6 were more likey among pregnant women who had good knowledge about anemia compare to pregnant women who had poor knowledge (AOR: 2.63, 95% CI 1.51, 4.59). Good knowledge of iron folate supplementation (AOR: 2.82, 95% CI 1.52, 5.23), four and above ANC visits (AOR: 6.97, 95% CI 3.25, 14.96), and current pregnancy anemia (AOR: 13.87, 95% CI 3.75, 51.35) were also significant predictors of adherence to iron folate supplementation for pregnant women (Table 3).

**Table 3:** Factors associated with iron folate supplementation adherence among pregnant women in West Dembia northwest Ethiopia 2018 (N = 348)

Characteristics	Adherence		COR (95% CI)	AOR (95% CI)
	Yes Number (%)	No Number (%)		
Knowledge about anemia				
Good knowledge	80 (40.0)	120 (60.0)	3.54 (2.25, 5.57)	2.63 (1.51, 4.59)*
Poor knowledge	104 (70.3)	44 (29.7)	1	1
Knowledge about iron folate supplementation				
Good knowledge	102 (42.7)	137 (57.3)	4.08 (2.46, 6.76)	2.82 (1.52, 5.23)*
Poor knowledge	82 (75.2)	27 (24.8)	1	1
Number of ANC visit				
≤ 2	105 (64.4)	58 (35.6)	1	1
3	66 (53.7)	57 (46.3)	1.56 (0.97, 2.52)	1.32 (0.78, 2.26)
≥ 4	13 (21.0)	49 (79.0)	6.82 (3.42, 13.61)	6.97 (3.25, 14.96)*
Previous anemia				
Yes	3 (9.7)	28 (90.3)	12.42 (3.70, 41.71)	13.87 (3.75, 51.35)*
No	181 (57.1)	136 (42.9)	1	1
Previous malaria				
Yes	7 (33.3)	14 (66.7)	2.36 (0.93, 5.99)	2.76 (0.91, 8.39)
No	177 (54.1)	150 (45.9)	1	1
Monthly income				
< 37$	17 (54.8)	14 (45.2)	1	1
38–111$	109 (42.2)	149 (57.8)	0.60 (0.28, 1.27)	0.89 (0.37, 2.13)
> 111$	38 (64.4)	21 (35.6)	1.49 (0.61, 3.61)	1.21 (0.43, 3.40)

*ANC* antenatal care

* Statistically significant at *p* < 0.05 after being adjusted for other variables, 1: reference

### Discussion

In Ethiopia, all women who seek ANC services from Government health facilities receive iron folate tablets for free. In this study, 52.9% (95% CI (47.7, 58.0)) of the pregnant women adhered to iron folate supplementation. This finding was higher than study in Tigray Region, which reported 28.9% respectively [8]. The finding was however lower than 60.9 and 70.6% detected in Addis Ababa [15], and Mizan Aman town [16], Ethiopia, respectively. The justifications for the differences are variations in socio-economic status, descripance in the accessments of health care facilities, dissimilarities in the study periods, and levels of awareness among pregnant women as well as in the availability of iron folate tablets.

In this study, the number of ANC visits was one of the significant predictors of adherence to iron folate supplementation of pregnant women. Women who had four and above ANC visits were significantly associated with adherence to iron folate supplementation compared to women who had less ANC visits. This finding was supported by those of studies done in Tigray Region, Ethiopia, and Indonesia [13, 17], respectively. The possible reason might be that when the number of ANC visits increases the opportunity for getting a sufficient number of iron folate tablets also increases. In addition, one of the essential nutrition action contact point is pregnancy. Therefore, during ANC visits, counseling about iron folate supplementation and advice to continue taking iron folate tablets is expected from health workers who give ANC services.

Pregnant women who had good knowledge of anemia were 2.63 times more likely to adhere to iron folate supplementation than their counterparts. This was in line with studies done in Mecha district, west Amhara [18] and rural districts of Ethiopia [19]. This might be due to the fact that when a pregnant woman has knowledge she tends to take iron folate tablets during pregnancy [20].

In this study, knowledge about iron folate supplementation was positively associated with adherence, which was supported by different studies, like in the rural districts of Ethiopia [19], for example, in west Amhara [18] and a study in southeastern Nigeria [21]. This may be probably became pregnant women who have awareness about the importance of iron folate supplementation during pregnancy are more likely to take the tablets than women who have no knowledge.

This study showed that women who had history of anaemia during the then pregnancy were more likely to adhere compared to women who no such knowledge. This finding was similar to those of studies done in Western Amhara, Ethiopia [18] and Tanzania [22]. The reason might be that women who had anemia history took more tablets for fear of side effects, and that health providers gave more emphasis to anaemic clients than to non-anaemic ones.

In this study, the main reason for non-adherence was forgetfulness. Like the results of India [23], a rural district of Ethiopia [19], and south eastern Nigeria [21]. The possible explanation might be that health professionals did not appropriately council about the benefities, side effects, and how to memorize the time for taking iron folate.

### Conclusion

This research showed the level of adherence of pregnant women to iron folate supplementation. The study indicated that there was a low level of adherence to iron folate supplementation among pregnant women who had less than four ANC visits, anemic during the then pregnancy, had poor knowledge about anemia and iron folate supplementation. Therefore, prevention of prenatal anemia, improving knowledge of women about anaemia and iron folate supplementation, and increasing the coverage of ANC services is essential to increase adherence to iron folate supplementation.

### Limitations of the study

The limitation of this study might be under or overestimation of the proportion of adherence and the health conditions of pregnant women because the data were self reported.

## Abbreviations

ANC: antenatal care
EDHS: Ethiopian Demographic and Health Survey
WHO: World Health Organization
